# Gene Expression Profiling of Iron Deficiency Chlorosis Sensitive and Tolerant Soybean Indicates Key Roles for Phenylpropanoids under Alkalinity Stress

**DOI:** 10.3389/fpls.2018.00010

**Published:** 2018-01-19

**Authors:** Brian M. Waters, Keenan Amundsen, George Graef

**Affiliations:** Department of Agronomy and Horticulture, University of Nebraska-Lincoln, Lincoln, NE, United States

**Keywords:** soybean, iron deficiency chlorosis, IDC, phenylpropanoid, transcriptomics, alkalinity stress

## Abstract

Alkaline soils comprise 30% of the earth and have low plant-available iron (Fe) concentration, and can cause iron deficiency chlorosis (IDC). IDC causes soybean yield losses of $260 million annually. However, it is not known whether molecular responses to IDC are equivalent to responses to low iron supply. IDC tolerant and sensitive soybean lines provide a contrast to identify specific factors associated with IDC. We used RNA-seq to compare gene expression under combinations of normal pH (5.7) or alkaline pH (7.7, imposed by 2.5 mM bicarbonate, or pH 8.2 imposed by 5 mM bicarbonate) and normal (25 μM) or low (1 μM) iron conditions from roots of these lines. Thus, we were able to treat pH and Fe supply as separate variables. We also noted differential gene expression between IDC sensitive and tolerant genotypes in each condition. Classical iron uptake genes, including ferric-chelate reductase (FCR) and ferrous transporters, were upregulated by both Fe deficiency and alkaline stress, however, their gene products did not function well at alkaline pH. In addition, genes in the phenylpropanoid synthesis pathway were upregulated in both alkaline and low Fe conditions. These genes lead to the production of fluorescent root exudate (FluRE) compounds, such as coumarins. Fluorescence of nutrient solution increased with alkaline treatment, and was higher in the IDC tolerant line. Some of these genes also localized to previously identified QTL regions associated with IDC. We hypothesize that FluRE become essential at alkaline pH where the classical iron uptake system does not function well. This work could result in new strategies to screen for IDC tolerance, and provide breeding targets to improve crop alkaline stress tolerance.

## Introduction

Iron (Fe) is an essential micronutrient for plants. Iron deficient plants increase root Fe uptake capacity by one of two strategies (Kobayashi and Nishizawa, 2012); grasses use a chelation-uptake strategy, while non-grass monocots and dicots employ what is classically considered a reduction-uptake strategy. In the reduction-uptake strategy, Fe deficient roots acidify the rhizosphere with H^+^-ATPases, encoded by the *AHA* or *HA* genes, they reduce ferric iron [Fe(III)] to ferrous iron [Fe(II)] with ferric-chelate reductase (FCR) proteins encoded by the *FRO* genes, and they take up iron with transporters encoded by *IRT* genes (Eide et al., 1996; Robinson et al., 1999; Santi and Schmidt, 2009).

While Fe is usually abundant in soil, its solubility is low in alkaline soils, which can lead to iron deficiency chlorosis (IDC), a functional Fe deficiency where leaves are yellow instead of green (Mengel, 1994). Plant species that use the reduction-uptake strategy, such as soybean [*Glycine max* (*L*.)*Merr*.], tend to be more sensitive to alkaline soils than grasses that use the chelation-uptake strategy. Approximately 30% of the earth has alkaline soils (Chen and Barak, 1982), including parts of the North-Central region of the U.S. In this region IDC is an important factor that limits soybean productivity and leads to yield losses of $260 million annually (Hansen et al., 2004; Peiffer et al., 2012). Soil alkalinity is primarily due to bicarbonate (Bic) and carbonate ions. Alkaline pH and Bic decrease solubility, and in turn availability of Fe, and may also inhibit Fe uptake gene expression and/or function (Lucena et al., 2007; Hsieh and Waters, 2016). IDC can be induced in hydroponic studies by buffering the nutrient solution at alkaline pH using Bic (Chaney et al., 1992). IDC can be induced in Bic-containing alkaline solutions even with Fe sources that remain available [e.g., Fe(III)-EDDHA, Halvorson and Lindsay, 1972] at quantities of soluble Fe that would be adequate in the ideal pH range. Thus, the effects of alkalinity extend beyond simply decreasing Fe availability. Plants that are Fe deficient from low Fe supply at normal pH also have chlorotic leaves, but it is not clear that this chlorosis is equivalent to IDC under alkaline conditions. Most plant Fe nutrition studies have induced Fe deficiency by limiting Fe supply in the normal, mildly acidic pH range of 5–6. Many studies have cataloged genes that are upregulated in roots in response to Fe deficiency in *Arabidopsis thaliana* and other plant species (Colangelo and Guerinot, 2004; Dinneny et al., 2008; García et al., 2010; Yang et al., 2010; Ivanov et al., 2012; Stein and Waters, 2012). Only a few transcriptomic, proteomic, or metabolomic studies have included alkaline pH in combination with low Fe supply to induce IDC (Rellán-Álvarez et al., 2010; Rodríguez-Celma et al., 2011a; Rodríguez-Celma et al., 2013; Schmidt et al., 2014). However, these studies did not include all combinations of normal and alkaline pH with high and low Fe supply. Thus, it is not clear whether results of Fe deficiency studies can be applied to IDC studies aimed at improving IDC tolerance in alkaline soils. Our first objective was to treat Fe supply and nutrient solution pH as separate variables to determine whether alkaline IDC conditions and low Fe supply affect soybean root gene expression in the same manner.

As discussed above, IDC effects are likely to be more complicated than Fe deficiency from low Fe supply. Soybean lines overexpressing the *A. thaliana FRO2* gene were constructed to improve Fe uptake (Vasconcelos et al., 2006). These lines had increased leaf and root Fe concentration, and increased *FRO2* expression and FCR activity in hydroponic nutrient solution at pH 5.5 (Vasconcelos et al., 2006). However, in alkaline soil field trials these lines did not have improved IDC tolerance (Kocak, 2014), suggesting that some factor other than FCR activity is the rate limiting step for Fe uptake under alkaline conditions, and/or the overexpressed FCR activity was not maintained in the alkaline soil conditions. In other dicot species, high Bic concentrations inhibited induction of FCR activity (Romera et al., 1992) and expression of Fe uptake genes (Lucena et al., 2007) in Fe deficient plants. However, plants supplied with Fe responded to low concentrations of Bic as if they were Fe deficient, with increased FCR activity and *FRO1* gene expression (Hsieh and Waters, 2016). Although Fe uptake responses were upregulated by Bic, Fe accumulation was impaired (Hsieh and Waters, 2016), again suggesting that the reduction-uptake components of this system are not the rate-limiting factors for Fe uptake in alkaline conditions. Thus, our second objective was to use soybean root gene expression data to improve our understanding of the physiological and biochemical responses to low Fe supply and alkaline conditions. One of the key findings in this work was upregulation of the phenylpropanoid pathway by Fe deficiency and alkalinity. In *A. thaliana*, this pathway produces phenolic root exudate compounds in the coumarins class that are involved in Fe uptake in alkaline conditions (Fourcroy et al., 2014; Schmid et al., 2014; Schmidt et al., 2014).

There is substantial variation for IDC tolerance in soybean (Rodriguez de Cianzio et al., 1979). IDC has been studied from plant breeding and genetics approaches, such as quantitative trait loci (QTL) mapping in biparental populations (Lin et al., 1997, 2000; Charlson et al., 2005) or introgression lines (Peiffer et al., 2012). Genome-wide association mapping studies (GWAS) were used to identify QTL associated with IDC (Wang et al., 2008; Mamidi et al., 2011, 2014). However, translating results from these genetic studies to specific genes for IDC tolerance has been challenging, since many QTL for IDC have small genetic effects, do not replicate robustly, or cover large genomic regions. Even without knowledge of specific genes that provide IDC tolerance, plant breeders can develop soybean lines with IDC tolerance (Prohaska and Fehr, 1981), however, a further understanding of the molecular aspects of IDC tolerance could accelerate breeding efforts by allowing breeders to target specific genes. Our third objective was to determine gene expression differences between IDC tolerant and susceptible varieties to aid in identifying specific factors that provide IDC tolerance.

The long-term goal of this research is to develop new IDC tolerant soybean varieties. A more complete understanding of IDC and Fe deficiency will increase our fundamental understanding of plant biology and lead to new strategies for improving crop production on Fe-deficiency prone, alkaline soils. This knowledge could also allow manipulation of these mechanisms to increase Fe concentrations in edible portions of plants for biofortification of foods.

## Materials and methods

### Plant materials and growth conditions

**Figure 1:**
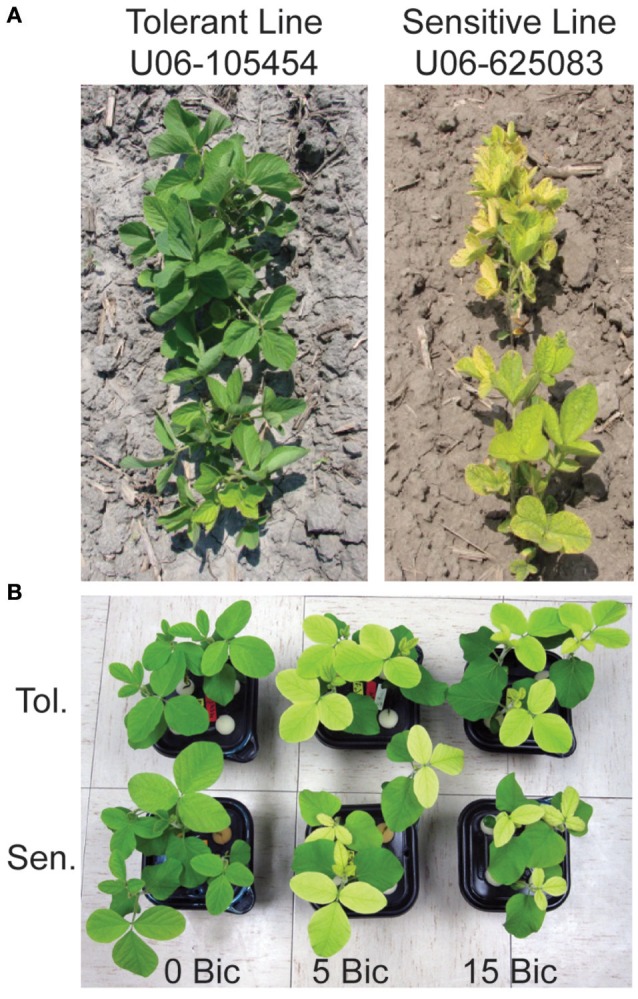
Soybean lines used in this study at the V3 stage. **(A)** IDC tolerant line U06-454 and IDC sensitive line UO6-625083 in an alkaline field in North Bend, Nebraska, USA. **(B)** The IDC tolerant and sensitive lines in hydroponic solution with low Fe supply [1 μM Fe(III)-EDDHA] and 0, 5, or 15 mM sodium bicarbonate at 12 d after planting.

The IDC tolerant line, U06-105454, shows extreme resistance to IDC on high-pH soils in Nebraska (Figure 1A). This line was selected from an inter-mated population developed from 10 original soybean parental lines selected on yield and better-than-average response to IDC. The line U06-105454 was selected after 10 cycles of recurrent selection for improved IDC tolerance based on foliar symptoms at the V3 stage. The IDC susceptible line, U06-625083, is a high-yield breeding line. Details about the genetic background of these lines and their IDC scores can be found in Kocak (2014).

A hydroponic system was developed to distinguish between IDC tolerant and sensitive lines to study root responses to iron deficiency and alkaline stress. Sodium bicarbonate (Bic) was added to the nutrient solution up to 15 mM, as indicated in each figure, to mimic high carbonate, alkaline soils, and to buffer the pH in the alkaline range. Seeds were germinated in germination paper soaked with 0.1 mM CaSO_4_ and incubated in the dark at 23°C for 7 days. Seedlings were transferred to black containers (two seedlings per tub) with 800 mL of nutrient solution made with 1.5 mM KNO_3_, 0.8 mM Ca(NO_3_)_2_, 0.3 mM (NH_4_)H_2_PO_4_, 0.2 mM MgSO_4_, 25 μM CaCl_2_, 25 μM H_3_BO_3_, 2 μM MnCl_2_, 2 μM ZnSO_4_, 0.5 μM Na_2_MoO_4_, 0.1 μM CuSO_4_, and 1 mM MES buffer (pH 5.5). For initial characterization, FCR activity, and RNA-seq gene expression, Fe was added as Fe(III)-EDDHA in experiments. The Fe(III)-EDDHA is stable at the mildly acidic and alkaline pH used in this study (Chaney et al., 1972; Halvorson and Lindsay, 1972). Plants were pretreated on either low (1 μM) or normal (25 μM) Fe for 6 d, then Bic was added to the solutions at the concentrations indicated for an additional 4 d. Final solution pH (at the end of each experiment) for each Bic concentration was: 0, 5.8 ± 0.59; 1.0 mM, 7.0 ± 0.34; 2.5 mM, 7.7 ± 0.16; 5 mM, 8.2 ± 0.15; 15 mM, 8.8 ± 0.19. For whole plant mineral analysis and fluorescent root exudate (FluRE) compound measurement experiments, plants were grown for 12 d in the solution as above, except that Fe was added as FeSO_4_, and Bic was included in the solutions for the entire 12 d.

### Ferric chelate reductase activity and mineral analysis

Ferric chelate reductase activity was measured using whole roots of individual 10 d old soybean plants after 4 d of Bic treatment. Roots were excised, rinsed in deionized water, and submerged in 50 ml assay solution (1 mM MES buffer, pH 5.5, 150 μM Fe(III)-EDTA, and 200 μM ferrozine [Sigma]) for 60 min. Ferrozine-Fe(II) was measured by absorbance at 562 nm and reduced Fe was calculated using the extinction coefficient of 28.16 mM cm^−1^. Chlorophyll of the first trifoliate leaf was determined using a SPAD-502 chlorophyll meter (Minolta) by taking the average of nine measurements per leaf (three per leaflet). Results are means from six individual runs of the same experiment. Significant differences between control and treatments, and between lines at each treatment were determined by *t*-test.

For leaf Fe concentration analysis, the first trifoliate leaf was collected from each of six plants grown in Fe(III)EDDHA with the final 4 d providing Bic treatments as above. For whole-plant Fe content analysis, four plants per treatment, grown as described for FluRE analysis, were dissected into roots, first trifoliate leaf, and the remainder of the plant (stem, unifoliate leaves, and cotyledons). Tissues were dried at 70°C in a drying oven. After measuring DW, tissue samples were digested in concentrated nitric acid/hydrogen peroxide with stepwise heating at 100, 125, 150, and 165°C to dryness, and then resuspended in 5 ml 1% HNO_3_ (Guttieri et al., 2015). Iron concentration was quantified in resuspended digests using an Agilent 7500cx ICP-MS (Agilent Technologies Inc., Santa Clara, CA) with Ar carrier and a He collision cell at the University of Nebraska Redox Biology Center Proteomics and Metabolomics Core Facility. Iron content of each plant part was calculated by multiplying tissue Fe concentration by sample DW, and total plant Fe content was calculated by the sum of all parts. Significant differences between control and treatments, and between lines at each treatment were determined by *t*-test.

### RNA sequencing

The IDC tolerant and susceptible lines were grown as described above with 1 μM or 25 μM Fe(III)EDHA for 10 d in solutions with 0, 2.5, or 5 mM NaH_2_CO_3_ for the last 4 d. RNA was isolated from roots using the Plant RNeasy kit (Qiagen). RNA-seq was performed at the University of Nebraska Medical Center Next Generation Sequencing Core Facility using an Illumina HiSeq 2000 instrument. Barcoded libraries were constructed from 3 μg of root total RNA, with three biological replicate libraries per treatment. Replicates were run in separate lanes, with a total of six samples from different treatments in each lane. In total, 414,801,800 RNA-seq paired end 50 bp sequencing reads were obtained from the sequencing facility following initial quality checks. Trimmomatic (Bolger et al., 2014) removed Illumina sequencing artifacts (ILLUMINACLIP) and poor quality reads using the following parameters LEADING:3 TRAILING:3 SLIDINGWINDOW:4:15 MINLEN:36. Only 926 additional reads were removed, leaving an average of 11.8 million pairs of surviving reads per sample. The Williams 82 transcripts (Wm82.a2.v1) were downloaded from SoyBase (https://www.soybase.org/) and used as a reference for read mapping. The trimmed reads were mapped to the reference and differential gene expression was inferred between all pairwise sample-conditions using the Tuxedo pipeline (Trapnell et al., 2012). Venn diagrams were made using a tool found at http://bioinformatics.psb.ugent.be/webtools/Venn/. Pairwise comparisons were made within each genotype between RNA sequenced from roots of plants grown in control (25 μM Fe, 0 Bic) conditions and in complete solution (25 μM Fe) with Bic at 2.5 and 5 mM, and between control and low Fe (1 μM Fe) without (0 Bic) or with 2.5 or 5 mM Bic. Pairwise comparisons were made between genotypes in each combination of Fe and Bic supply. Genes were considered to be differentially expressed if they had a value in the treatments greater than or equal to the absolute value of the log2 control value. The full results of these pairwise comparisons are presented in **Supplementary Tables** as indicated in Results and Discussion. If a gene was differentially expressed in one treatment, we show the expression fold change values for all treatments and both genotypes in the tables, for completeness. The short reads are available to the soybean genomics community as NCBI BioProject: PRJNA389118.

### Fluorescent root exudate and extract measurements

Soybean plants were grown in 800 ml nutrient solution as described above, with or without 2.5 mM Bic, at 1 or 20 μM FeSO_4_ for 14 d. Fluorescence of the nutrient solution was used to estimate the quantity of exuded fluorescent compounds. Nutrient solution fluorescence was determined in 200 μL aliquots in a BioTek Synergy 2 microplate reader with excitation at 360 nm and detection at 460 nm with 40 nm windows and a sensitivity setting of 50. Total fluorescence units per container were calculated by multiplying by solution volume, and total fluorescence units per plant were calculated by dividing fluorescence units per container by root FW. Significant differences between control and treatments, and between lines at each treatment were determined by *t*-test.

## Results and discussion

Previous studies of soybean root gene expression in response to Fe deficiency or alkalinity used different genetic backgrounds, conditions and/or gene expression technologies than we did in this study. The current study treated Fe supply and solution pH as separate variables to gain a better understanding of the specific effects of those treatments. The seedlings for RNA-seq were exposed to treatments in hydroponics for 4 d at moderate Bic concentrations (2.5 or 5 mM), which resulted in a more severe chlorosis in the IDC sensitive line than in the IDC tolerant line (Figure 1B). In other studies, RNAseq was used to study early responses at 12 and 24 h of Fe deficiency at pH 7.8 (Moran Lauter et al., 2014). Of the 489 differentially expressed genes in roots in the previous study, only 144 overlapped with the 5,288 differentially expressed genes in roots in our study, suggesting that gene expression at early time points in response to IDC conditions may differ significantly from seedlings with a longer-term exposure. Since IDC symptoms in the field develop over days to weeks, we used treatments with a long enough duration to begin to cause leaf chlorosis that would distinguish the IDC sensitive and tolerant lines. Previous molecular-scale IDC studies have compared two isogenic lines of soybean, Clark and Isoclark, with microarrays (O'Rourke et al., 2009) or RNAseq (Peiffer et al., 2012) in Fe-replete or Fe-limiting solutions that were buffered at pH 7.8. The lines compared in our study resulted from a recurrent selection strategy from 10 parental lines with selection for leaf chlorosis (or lack thereof) over 11 cycles, resulting in genetically diverged lines. Thus, the genetic underpinnings of the differences in IDC tolerance between the lines we used are likely due to multiple loci.

### Physiological characterization

**Figure 2:**
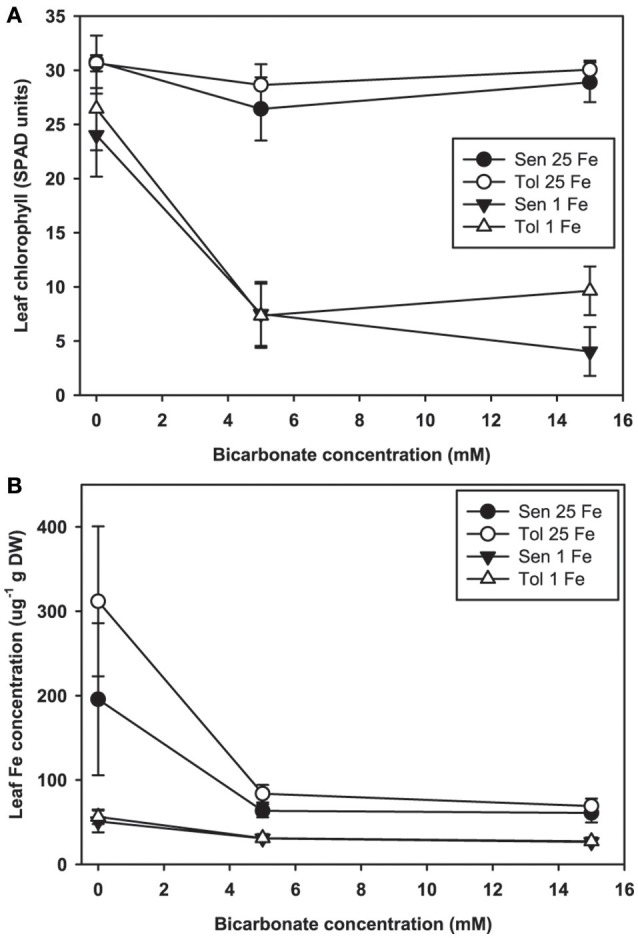
Leaf chlorophyll and iron (Fe) concentration in IDC tolerant and sensitive soybean plants. **(A)** chlorophyll (in SPAD units) and **(B)** Fe concentration in the first trifoliate leaf. Plants were pretreated with 1 or 25 μM Fe(III)-EDDHA for 6 d then treated with 1 or 25 μM Fe(III)-EDDHA and 0, 5, or 15 mM sodium bicarbonate for 4 d. At low Fe supply, Bic treatment resulted in statistically significantly lower leaf chlorophyll (*p* < 0.05), and the genotypes were significantly different at 15 mM Bic. At both Fe levels, addition of Bic resulted in statistically significantly lower leaf Fe concentration (*p* < 0.05), but there were no differences between genotypes.

Leaf chlorosis occurred only in plants treated with both low Fe supply and alkaline nutrient solution. In our hydroponic system at low Fe(III)-EDDHA supply without Bic, leaves of both the IDC sensitive and IDC tolerant lines remained green (Figure 2A). With normal Fe supply, addition of Bic at up to 15 mM did not result in leaf chlorosis, however, at low Fe supply, 5 mM Bic treatment resulted in chlorosis. Bic treatment resulted in greatly decreased leaf Fe concentration (Figure 2B) in both the IDC tolerant and sensitive lines and at both Fe supply levels. Although the leaves were green, leaf Fe concentration decreased from 200 to 300 μg/g without Bic to ~60 μg/g with Bic. Bic treatment decreased leaf Fe concentration more than low Fe supply alone, but a combination of Bic and low Fe resulted in the lowest Fe concentrations. This result suggests that Fe uptake was inhibited at alkaline pH, in agreement with previous soybean studies (Coulombe et al., 1984; Fleming et al., 1984), but it was not inhibited to the extent that leaves developed chlorosis.

**Figure 3:**
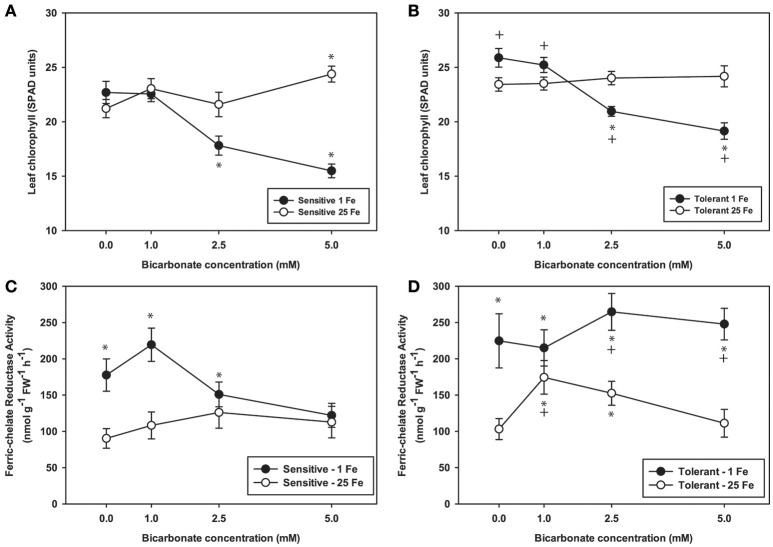
Leaf chlorophyll and root ferric-chelate reductase activity in IDC tolerant and sensitive soybean plants. Plants were pretreated with 1 or 25 μM Fe(III)-EDDHA for 6 d then treated with 1 or 25 μM Fe(III)-EDDHA and 0, 1, 2.5, or 5 mM sodium bicarbonate for 4 d. First trifoliate leaf chlorophyll (in SPAD units) in **(A)** IDC sensitive plants and **(B)** IDC tolerant plants. Root FCR activity in **(C)** IDC sensitive plants and **(D)** IDC tolerant plants. ^*^ indicates a significant difference (*p* < 0.05) between treatment and control (25 Fe, 0 Bic). + in **(B)** and **(D)** indicates a significant difference between the tolerant and sensitive lines in that treatment.

To further explore effects of alkaline pH on Fe uptake processes, we measured leaf chlorophyll and tested FCR activity from plants grown at Bic concentrations up to 5 mM (Figure 3), since it was within this range that most of the chlorophyll and leaf Fe concentration decrease occurred (Figure 2). Over this range, again chlorosis only occurred in plants with both low Fe supply and Bic treatment. At 2.5 or 5 mM Bic and low Fe, the susceptible line had a greater decrease in chlorophyll than the tolerant line (Figures 3A,B), consistent with field results (Figure 1). For plants grown in solutions without Bic (pH 5.9), both lines had higher FCR activity at low Fe supply relative to normal Fe, reflecting the expected upregulation of Fe uptake responses (Kobayashi and Nishizawa, 2012) by this mild Fe deficiency. As Bic supply was increased to 2.5 or 5 mM, the susceptible line's FCR activity was no longer stimulated at low Fe supply relative to normal Fe supply (Figure 3A). However, the tolerant line maintained higher FCR activity at low Fe supply as Bic increased (Figure 3B). Thus, the IDC tolerant line's FCR activity was not as susceptible to alkaline stress as FCR activity in the sensitive line, although this activity was apparently not sufficient to maintain Fe uptake (Figure 2). This result may explain why *AtFRO2* overexpressing soybean plants did not have improved IDC tolerance on alkaline soils (Kocak, 2014).

### Gene expression profiling

**Figure 4:**
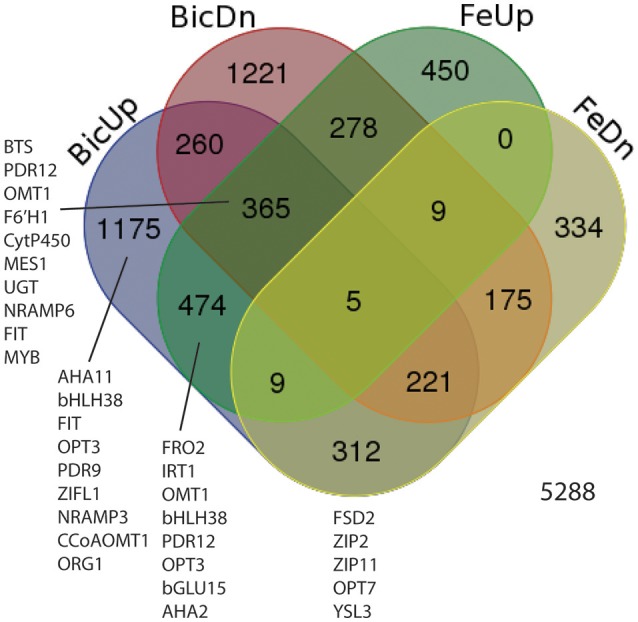
Venn diagram showing differentially expressed genes (DEGs) in roots of IDC sensitive or tolerant soybean lines (genotypes combined). BicUp, DEGs upregulated by 2.5 and/or 5 mM bicarbonate treatment relative to 0 bicarbonate; BicDn, DEGs downregulated by bicarbonate treatment; FeUp, DEGs upregulated by low Fe (1 μM) treatment relative to normal Fe (25 μM) treatment; FeDn, DEGs downregulated by low Fe (1 μM) treatment. Numbers represent number of elements (genes) in each set. Genes of interest in each set are listed near the set, and correspond to highlighted genes in Supplementary Table 2.

Based on the results above, transcripts were quantified by RNA-seq from roots from IDC sensitive and tolerant plants grown at low Fe or normal Fe supply, with 0, 2.5, or 5 mM Bic. Our first goal was to determine whether, in soybean as a species, Fe deficiency and alkalinity are equivalent in terms of specific genes that are induced or repressed. As such, we combined results from both genotypes (Figure 4) to capture all DE genes in a larger set that was then observed in detail to determine each genotype's response. Of 5,288 differentially regulated genes (Supplementary Table 1), 474 were upregulated by both Fe deficiency and Bic treatment, while 175 were downregulated by both Fe deficiency and Bic treatment. A greater total number of genes were differentially regulated by only Bic or Fe deficiency than were regulated by both stresses. Many of the classical Fe responsive genes (*FRO2, IRT1, OPT3, AHA2*, and *bHLH38*) were upregulated by both Fe deficiency and alkaline stress (Figure 4, Supplementary Table 2). However, *FIT* and *NRAMP6* were among the 365 genes that were upregulated by Fe deficiency and both upregulated and downregulated by alkaline stress, depending on genotype and treatment. Homologs of some genes that have been regulated by Fe deficiency in previous studies (Colangelo and Guerinot, 2004; García et al., 2010; Yang et al., 2010; Stein and Waters, 2012) were only upregulated by Bic treatment (*FIT, bHLH38, OPT3, NRAMP3, ORG1*). Some metal homeostasis genes were downregulated by Fe deficiency and upregulated by Bic treatment: *FSD2, ZIP2, ZIP11, OPT7*, and *YSL3*. Thus, while many of the Fe uptake genes were regulated similarly by Fe deficiency and alkaline stress, there were many genes that did not respond equivalently, suggesting that alkalinity stress stimulates expression of Fe uptake genes, but also has other effects.

We compared differential expression of soybean homologs of Arabidopsis classical Fe deficiency response genes *FRO2, IRT1, AHA2*, and *FIT* across the soybean lines (Table 1). At low Fe supply the IDC sensitive line had significantly increased expression of the four Fe uptake genes, while the tolerant line had increased expression of only *FRO2*, suggesting that the IDC sensitive line was also more sensitive to Fe deficiency. However, as Bic increased and the pH became more alkaline, the tolerant line upregulated expression of *FRO2, IRT1*, and *FIT*, while their expression decreased in the sensitive line. While this result does not necessarily indicate that the proteins produced from these Fe uptake transcripts are functional in alkaline conditions, it does suggest that, like with FCR activity results (Figure 3), expression of Fe uptake genes in the sensitive line was inhibited by Bic to a greater extent than in the tolerant line. Iron uptake gene expression was also stimulated by alkaline stress at normal Fe supply, with significant upregulation of *FRO2, IRT1, AHA2*, and *FIT* in the tolerant line, and upregulation of *FRO2* and *IRT1* in the sensitive line. Since the Fe(III)-EDDHA is pH stable (Halvorson and Lindsay, 1972), this response is not due to decreased Fe availability to the roots. The response of classical Fe uptake genes indicates that both low Fe supply and alkaline pH stress induces Fe uptake responses at the molecular level. At this point it is unclear whether Fe uptake gene upregulation is due to the plant directly sensing alkalinity at the roots, or whether the upregulation results from a leaf-originated or local root signal that is stimulated by low Fe concentration in the plant tissues (Figure 2) resulting from alkalinity inhibited Fe uptake. Results from cucumber indicated that when alkaline stress is combined with Fe deficiency the normal Fe deficiency sensing in the leaf is blocked, potentially resulting in loss of shoot-to-root signal of plant Fe status (Hsieh and Waters, 2016).

**Table 1:** Comparison of differentially expressed genes for classical iron deficiency response, riboflavin synthesis, and phenylpropanoid synthesis from *Medicago truncatula* (M.t.), *Cucumis melo* (C.m.), *Arabidopsis thaliana* (A.t.), and soybean, *Glycine max* (G.m.).

						**Flavin producers**		**Coumarin producer**	Soybean: non-flavin producer^*^									
**A.t. gene**	**Gene name**	**C.m. gene**	**M.t. gene**	**G.m. gene**	**Description**	**M.t. -Fe^†^**	**C.m. -Fe^‡^**	**A.t. -Fe^†^**	**1 Fe Sen**	**1 Fe 2.5 Bic Sen**	**1 Fe 5 Bic Sen**	**25 Fe 2.5 Bic Sen**	**25 Fe 5 Bic Sen**	**1 Fe Tol**	**1 Fe 2.5 Bic Tol**	**1 Fe 5 Bic Tol**	**25 Fe 2.5 Bic Tol**	**25 Fe 5 Bic Tol**
**CLASSICAL Fe RESPONSE**																		
AT1G01580	FRO2	MELO3C011744	Medtr7g038510	Glyma.07G067700	Ferric-chelate reductase	5.5	4.3	5.9	5.3	5.5	4.0	4.0	5.1	2.8	7.2	7.2	5.1	3.0
AT4G19690	IRT1	MU57707	Medtr4g083570	Glyma.07G223200	Fe(II) transport protein	3.0	1.4	5.8	3.7	4.1	3.3	2.5	3.5		6.3	6.4	4.0	2.6
AT4G30190	AHA2	MELO3C017859	Medtr4g127710	Glyma.13G097900	H(+)-ATPase	−0.8		0.9	1.2							1.1		0.8
AT2G28160	FIT	MELO3C026952	contig_81683_1	Glyma.11G192800	Transcriptional regulator of iron uptake	7.4	1.9	2.1	1.8						2.6	2.9	1.6	0.9
AT3G56970	bHLH038	MELO3C019065	Medtr7g090410	Glyma.03G130600	Basic helix-loop-helix (bHLH) DNA-binding superfamily	2.4		2.1	2.1	3.4	3.3		1.4		1.6	1.2		
AT3G56970	bHLH038	MELO3C019065	Medtr7g090410	Glyma.03G130400	Basic helix-loop-helix (bHLH) DNA-binding superfamily	2.4		2.1	2.1	3.4	3.3				5.7			
**RIBOFLAVIN SYNTHESIS PATHWAY**																		
AT5G64300	GCH,RIB1A1,RFD1	MELO3C024826	Medtr2g009270	Glyma.15G055700	ATGCH; 3,4-dihydroxy-2-butanone-4-phosphate synthase/ GTP cyclohydrolase II	5.0	4.1							1.1	0.9	1.2		0.9
At4g20960	PYRD	MU51870	Medtr4g119220	Glyma.07G071800	Diaminohydroxyphosphoribosylaminopyrimidine deaminase	4.3												
AT3G47390	PHS1,PYRR	MELO3C010048	Medtr7g080120	Glyma.18G242600	Cytidine/Deoxycytidylate deaminase family protein	4.3	3.3											
AT3G47390	PHS1,PYRR	MELO3C010049	Medtr7g080120	Glyma.18G242600	Cytidine/Deoxycytidylate deaminase family protein		2.7											
AT2G44050	COS1	MU59012	contig_50382_2	Glyma.08G087500	COS1 (COI1 SUPPRESSOR1); 6,7-dimethyl-8-ribityllumazine synthase	6.4	1.8											
AT2G20690	RIBC	MELO3C000760	contig_57647_1	Glyma.12G112900	Lumazine-binding family protein (Riboflavin synthase)	6.0												
**PHENYLPROPANOID SYNTHESIS PATHWAY**																		
AT3G12900		MU47597		Glyma.08G169100	2-Oxoglutarate (2OG) and Fe(II)-dependent oxygenase			9.2	5.5	4.9	4.1	2.4	3.8	2.2	5.1	5.1	1.8	1.6
AT3G13610	F6'H1	MU47597	Medtr3g043900	Glyma.07G124400	2-Oxoglutarate (2OG) and Fe(II)-dependent oxygenase	−0.8		3.3	7.0	5.7	4.8		4.1		6.8	6.6	2.6	1.5
AT3G13610	F6'H1	MU47597	Medtr3g043900	Glyma.03G096500	2-Oxoglutarate (2OG) and Fe(II)-dependent oxygenase	−0.8		3.3	5.3	4.8	3.4		2.7	2.9	7.2	6.9		2.6
AT4G31940	CYP82C4	MU54527		Glyma.04G035600	Cytochrome P450, family 82, subfamily C, polypeptide 4			7.5	4.7	4.0	3.0		2.1		8.7			
AT2G34500	CYP710A1	MELO3C007140		Glyma.15G095000	Cytochrome P450, family 710, subfamily A, polypeptide 1			−1.4	0.8					1.2	1.0	1.4		0.8
AT4G36220	CYP84A1, FAH1	MELO3C007884		Glyma.08G140600	Ferulic acid 5-hydroxylase 1			−1.2	2.2					1.1				

† *Differential gene expression (Fold Change) for Arabidopsis thaliana genes indicated in “A.t. gene” column or Medicago truncatula genes indicated in “M.t. gene” column. Data from Rodríguez-Celma et al. (2013)*.

‡ *Differential gene expression (Fold Change) for Cucumis melo genes indicated in “C.m. gene” column. Data from Waters et al. (2014)*.

* *Sub-column headings indicate iron (Fe) supply (1 Fe, 1 μM; 25 Fe, 25 μM Fe), bicarbonate (Bic) treatment (2.5 Bic, 2.5 mM; 5 Bic, 5 mM), and genotype (Sen, IDC sensitive line; Tol, IDC tolerant line). Numbers indicate fold change difference in gene expression from this study*.

We also used our RNA-seq data to gain insight into key physiological responses to alkalinity and Fe deficiency. Some of the genes that were most highly responsive to both Fe deficiency and alkaline stress were homologs of phenylpropanoid synthesis gene *AtF6'H1* (Supplementary Table 2), which are needed to synthesize FluRE such as scopoletin (Kai et al., 2008), that are important for Fe uptake under alkaline conditions in Arabidopsis (Fourcroy et al., 2014; Schmid et al., 2014; Schmidt et al., 2014). In addition to the *F6'H1* genes, other putative phenylpropanoid genes were strongly upregulated by Fe deficiency and/or alkaline stress in soybean roots (e.g., *CYP82C4, MES1, UGT73C1, OMT1, BGLU15, CCoAOMT1*; Figure 4, Supplementary Table 2). We also noted upregulation of two putative phenylpropanoid transporters, most similar to *AtPDR9* and *AtPDR12*. *AtPDR9* and *BGLU42* are necessary to secrete coumarins from roots under Fe deficiency (Fourcroy et al., 2014; Zamioudis et al., 2014).

Iron deficient dicots are known to increase production and efflux of either flavin compounds or fluorescent phenolic compounds, but not both within a single species (Cesco et al., 2010; Rodríguez-Celma and Schmidt, 2013). Iron deficient Arabidopsis increases production of phenolics of the coumarin class (Rodríguez-Celma et al., 2013; Schmid et al., 2014; Schmidt et al., 2014) by increasing expression of the phenylpropanoid pathway, and our results suggest that soybean has a similar response. To indicate whether soybean might increase flavin production under Fe deficiency or alkaline stress, we compared soybean riboflavin synthesis gene expression to expression from other plant species under Fe deficiency (Rodríguez-Celma et al., 2013; Waters et al., 2014). The riboflavin synthesis genes were upregulated by Fe deficiency in melon (*Cucumis melo*) and *Medicago truncatula*, known flavin producers (Welkie, 2000; Rodríguez-Celma et al., 2011b), but not in Arabidopsis or soybean. Both Arabidopsis and soybean had increased expression of phenylpropanoid synthesis genes in response to Fe deficiency, but melon and Medicago did not (Table 1). In soybean, these phenylpropanoid synthesis genes also responded to Bic treatment. A simplified diagram of the known and some presumed steps in coumarin synthesis and efflux is shown in Supplementary Figure 1, showing Arabidopsis genes and their soybean homologs. Many of these genes are upregulated by Fe deficiency at the protein and/or transcript level in Arabidopsis (Lan et al., 2010; Rodríguez-Celma et al., 2013), and soybean homologs for all of these genes were upregulated in one or more Fe deficiency or alkaline stress conditions (Supplementary Table 2). These results suggest that soybean upregulates synthesis of coumarins in response to Fe deficiency and alkaline stress, similar to Arabidopsis. It is worth noting that, like the classical Fe response genes, expression of potential phenylpropanoid synthesis genes tended to decrease in the IDC sensitive line as Bic supply increased, while expression increased or remained high in the IDC tolerant line (Supplementary Table 2), which is why some of the Fe upregulated genes were categorized as both Bic upregulated and Bic downregulated (Figure 4).

### Fluorescent root exudates

**Figure 5:**
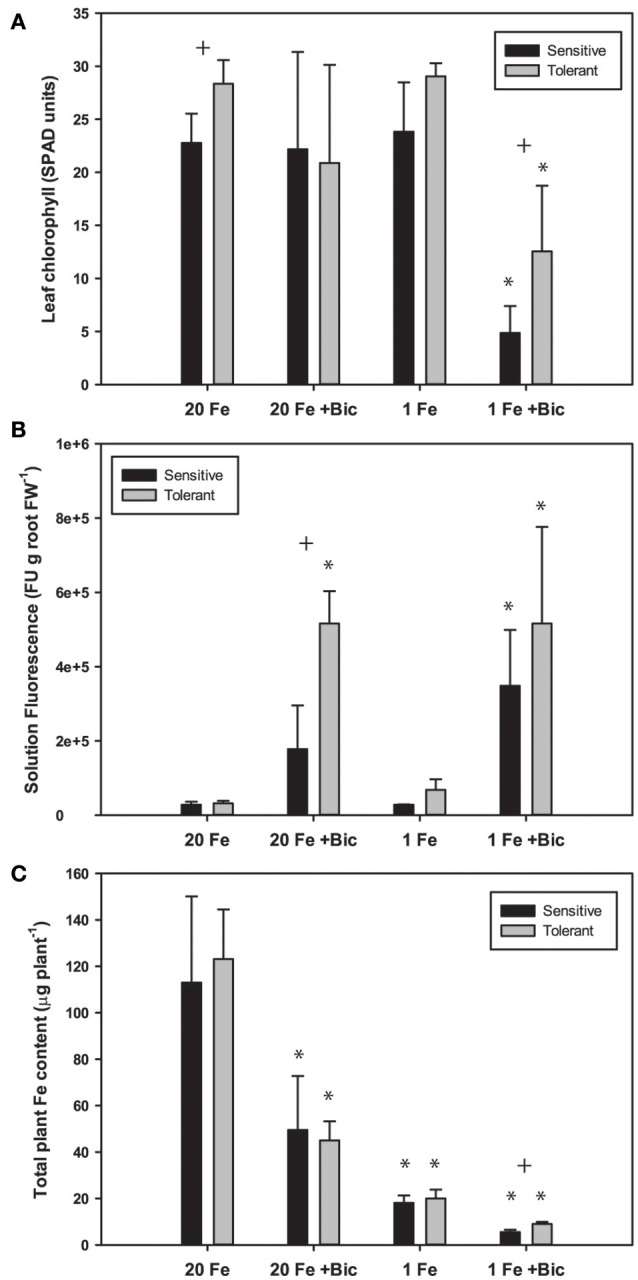
Leaf chlorophyll, nutrient solution fluorescence, and total plant Fe accumulation in IDC tolerant and sensitive soybean plants. Plants were treated with 1 or 20 μM FeSO_4_ and 0 or 2.5 mM sodium bicarbonate for 12 d. **(A)** leaf chlorophyll of first trifoliate leaf in SPAD units; **(B)** total fluorescence units of nutrient solution normalized to root FW; **(C)** Total plant Fe accumulation (content), in μg Fe per plant. ^*^ indicates a significant difference (*p* < 0.05) between treatment and control (20 Fe, 0 Bic). + indicates a significant difference between the tolerant and sensitive lines in that treatment.

Fe deficiency increases efflux of coumarins from Arabidopsis roots (Fourcroy et al., 2014; Schmid et al., 2014; Sisó-Terraza et al., 2016), and alkaline growth conditions result in greater coumarin production and exudation (Schmidt et al., 2014). Since coumarin compounds are fluorescent, fluorescence of the culture media is a good estimate of total coumarin root exudates (Schmid et al., 2014; Schmidt et al., 2014). We measured fluorescence of hydroponic solutions following growth of soybean plants in solutions with normal and low concentrations of FeSO_4_ and with a range of Bic. We used FeSO_4_ rather than Fe(III)EDDHA in these experiments because the non-chelated FeSO_4_ would have decreased availability at alkaline pH, and if the FluRE could assist with Fe availability they would be helpful in growth on this Fe source. Phenolic root exudates are important for Fe uptake in Strategy I species (Clemens and Weber, 2016). In both red clover and Arabidopsis, removing phenolics from recirculating nutrient solution resulted in increased severity of Fe deficiency (Jin et al., 2007; Fourcroy et al., 2014). Phenolic compounds are needed to access precipitated Fe in the apoplast of roots (Jin et al., 2007; Ishimaru et al., 2011) and have Fe chelating properties and Fe(III) reducing activity (Schmid et al., 2014; Schmidt et al., 2014). When plants were grown in hydroponics with Bic and FeSO_4_ as the Fe source, IDC tolerant lines had higher leaf chlorophyll (Figure 5A). In both the IDC sensitive and IDC tolerant lines, alkaline growth conditions resulted in greatly increased fluorescence (Figure 5B), and fluorescence was higher from the IDC tolerant line than for the IDC sensitive line, although the difference was not statistically significant.

Arabidopsis T-DNA lines with disruptions in genes required for synthesis or efflux of FluRE were more sensitive to Fe deficiency and had poor growth under alkaline conditions relative to wild-type plants; specifically for the *F6'H1* gene (Schmid et al., 2014; Schmidt et al., 2014), a 2-oxoglutarate (2OG) and Fe(II)-dependent oxygenase needed for coumarin synthesis (Kai et al., 2008), and *ABCG37/PDR9*, a coumarin efflux transporter gene (Fourcroy et al., 2014). Homologs of these genes were significantly upregulated in soybean roots (Table 1). If these coumarin compounds are important for Fe uptake at alkaline pH, then the higher FluRE in the solution of the IDC tolerant line could explain why it can grow better in alkaline, IDC-prone conditions. To test whether the IDC tolerant line that made greater quantities of FluRE had higher overall Fe accumulation than the IDC sensitive line, we grew the plants in hydroponics for 12 d, changed the Fe source to FeSO_4_ to more closely mimic soil conditions without synthetic chelators, and also began Bic treatments upon planting. Total plant Fe content was not different between IDC sensitive and tolerant lines (Figure 5C), even though the sensitive line had more severe leaf chlorosis under the low Fe with Bic treatment. Alkaline growth solution decreased Fe accumulation in both lines, while low Fe supply decreased Fe accumulation further. This result suggests that the difference in IDC sensitivity between these lines is not attributable to differences in overall Fe uptake, at least in hydroponic growth conditions. This finding suggests that the IDC tolerant line has higher Fe use efficiency than the sensitive line. We have observed genetic differences in Fe use efficiency in cucumber seedlings (Waters and Troupe, 2012), but the basis for these differences are not well-understood. However, our mineral analysis method can only detect bulk Fe content, and cannot detect symplastic/apoplastic partitioning, subcellular Fe localization, or other fine-scale details. Further studies could determine whether these two lines partition Fe differently within the plant organs and indicate the basis for differences in Fe use efficiency.

There was a difference between IDC tolerant and sensitive lines for whole-plant Fe accumulation in our hydroponic system in the low Fe +Bic treatment (Figure 5C), where FluRE may be important for Fe uptake. There may be an even more important role for FluRE compounds in alkaline soil (Clemens and Weber, 2016), which is more chemically complex than hydroponic solution. It is also possible that FluRE are involved in processes in addition to Fe uptake. Certain microorganisms or volatile compounds they produce can stimulate expression of Fe uptake responses (Zhang et al., 2009; Zamioudis et al., 2015; Zhou et al., 2016), including FluRE production (Zamioudis et al., 2014), and many of the genes that are induced by these microbes in Arabidopsis have homologs that are induced by Fe deficiency and or alkalinity stress in this study (Supplementary Table 3). These results together suggest that a possible role for these fluorescent compounds is in modification of the rhizomicrobiome (Badri et al., 2013; Gu et al., 2016), which could in turn improve soybean immunity to pathogens (Zamioudis and Pieterse, 2011), or influence uptake or availability of Fe or other mineral nutrients (Cesco et al., 2010).

Phenylpropanoid pathway genes are regulated by certain MYB transcription factors, such as *MYB58* and *MYB63* in Arabidopsis (Zhou et al., 2009). The MYB72 gene is upregulated by Fe deficiency in Arabidopsis roots (Colangelo and Guerinot, 2004; García et al., 2010; Yang et al., 2010; Stein and Waters, 2012). Roots of a *myb72* mutant did not increase production or efflux of FluRE at pH 7.0 relative to control conditions at pH 5.8, whereas WT roots did produce and secrete FluRE (Zamioudis et al., 2014). Overexpression of Arabidopsis *MYB58* and *MYB63* and sorghum *MYB60* transcription factors resulted in increased expression of the entire phenylpropanoid pathway and increased lignin synthesis in Arabidopsis and sorghum (Zhou et al., 2009; Scully et al., 2016). Additionally, overexpression of the *MYB72* gene in Arabidopsis led to upregulation of the entire phenylpropanoid pathway and increased expression of FluRE (Zamioudis et al., 2014). Several soybean *MYB* transcription factors were upregulated by Fe deficiency or alkaline stress in a manner consistent with upregulation of the phenylpropanoid synthesis genes. However, further experiments are required to determine which of these *MYB* genes are specifically required for regulation of FluRE in soybean.

### Genotypic differences and correspondence to QTL

**Figure 6:**
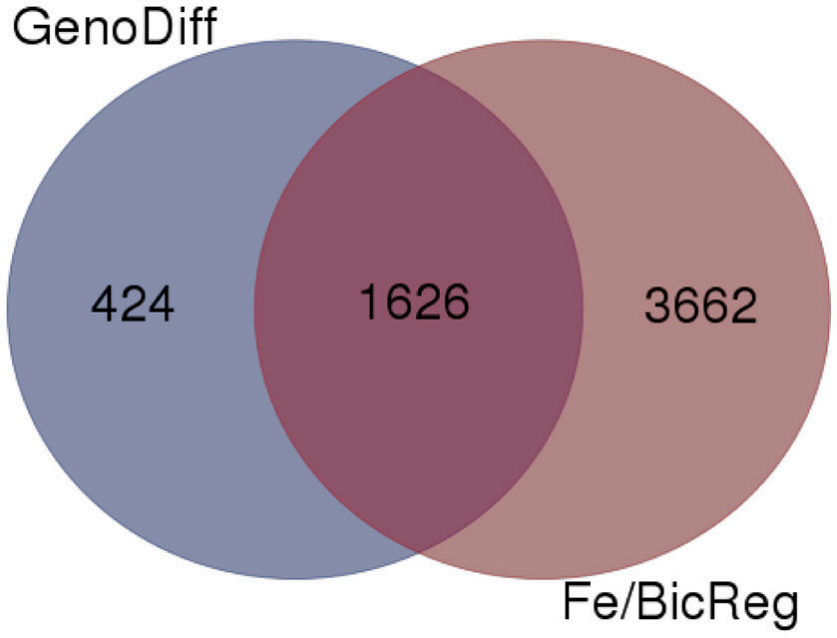
Venn diagram comparing genes with differential expression in a specific condition (low or normal Fe supply with 0, 2.5, or 5 mM bicarbonate) between IDC sensitive and tolerant lines (GenoDiff) to genes that responded to low Fe and/or bicarbonate in either line (Fe/BicReg). The 1,626 overlapping genes are featured in Supplementary Table 4.

To gain insight into the genetic aspects of IDC tolerance inherent in the two lines in this study, we compared transcript abundance in each of the lines in each treatment. Two thousand fifty genes had at least 1.0-fold difference in abundance (log2 scale) between IDC tolerant and sensitive lines, and 1,626 of these genes were also differentially regulated by Fe or alkalinity treatments (Figure 6). These 1,626 genes are shown along with the differential expression by treatment, in Supplementary Table 4. Many of the putative phenylpropanoid synthesis genes had higher transcript levels in the IDC tolerant line than in the IDC sensitive line.

To further compare our RNA-sequencing results with existing genetic IDC data, and determine whether phenylpropanoid genes are within previously mapped IDC QTL from biparental populations (Lin et al., 1997, 2000; Charlson et al., 2005; Wang et al., 2008; Mamidi et al., 2011, 2014; Kocak, 2014) and from GWAS studies (Wang et al., 2008; Mamidi et al., 2011, 2014) we aligned our RNA-seq results with QTL intervals. We used the information on Soybase to anchor the flanking genetic markers to the physical map, and genes within these intervals are shown in Supplementary Table 5. Notably, *FRO2* corresponded with a QTL on chromosome 7. *FRO2* was highly upregulated by Fe deficiency and Bic treatment, and had greater transcript abundance in one variety, depending on the treatment. Another Fe homeostasis gene, *BTS*, an E3 ligase that is involved in regulation of Fe deficiency responses (Long et al., 2010; Selote et al., 2015), corresponded with a QTL on chromosome 5. Genes for BTS-interacting transcription factors bHLH115 and bHLH105/IRL3 (Long et al., 2010) were upregulated under low Fe and low Fe +Bic treatments in both genotypes. One of the QTL intervals on Chr. 3 that explained 70% of the variation in an Anoka X A7 population (Lin et al., 1997) and that was introgressed in two IDC sensitive NILs (Severin et al., 2010; Peiffer et al., 2012) contained *bHLH38* (Glyma.03G130600). This gene was highly upregulated by Fe deficiency and alkaline stress, and was more abundant in the IDC tolerant line than in the sensitive line at 25 μM Fe with 2.5 mM Bic (Table 1, Supplementary Tables 2, 5). A previous IDC study (Peiffer et al., 2012) suggested that a 12 bp deletion in the *bHLH38* gene was the likely difference between the IDC tolerant (Clark) and sensitive (isoClark) isolines. That 12 bp variant in *bHLH38* was not present in our IDC tolerant and sensitive germplasm (data not shown), suggesting that the IDC sensitivity differences between our lines is not due to this particular variant. However, since the bHLH38 protein interacts with the FIT protein to control most Fe uptake responses in Arabidopsis (Yuan et al., 2008), the *bHLH38* gene may be a major factor for IDC tolerance.

Natural variation is present for coumarin synthesis in *A. thaliana*, with multiple QTL controlling this trait in plants grown without stress treatments (Siwinska et al., 2014). Variation for fluorescent exudate production in response to Fe deficiency and alkalinity stress conditions is also present in the soybean IDC sensitive and tolerant lines used in this study. Notably, the major chromosome 3 QTL region may also contain one of the *F'6H1* homologs (Glyma.03g096500) that was strongly upregulated by Fe deficiency and Bic treatment and was more abundant in the IDC tolerant line. Several other previously mapped IDC QTL intervals contained potential phenylpropanoid synthesis genes that were upregulated by Fe deficiency or alkaline stress, including a cytochrome P450 family gene (Glyma.04g035600). There are also *MYB* genes, which may be involved in regulating the phenylpropanoid synthesis pathway, in QTL regions on chromosomes 2, 4, 11, 14, 16, and 19. These results indicate that differences in coumarin production may underlie some of the IDC QTL.

## Conclusions

This study has indicated a correlation between FluRE quantity and IDC tolerance, and has indicated specific genes that may be involved in the regulation, synthesis, and efflux of these compounds. Further physiological studies to fully define the roles for FluREs in Fe deficiency and IDC tolerance will be helpful. A future direction will be to test whether FluRE quantities from other soybean varieties correlate with IDC tolerance. Our transcriptomic results for alkaline stress and Fe deficiency regulated genes can be a valuable resource for other researchers to cross-reference to genetic studies. It will be useful to extend the study of FluRE to linkage mapping populations to further understand genetic control of FluRE production, and to determine whether FluRE contribute to IDC tolerance. The IDC tolerant and susceptible parents from this study were used to develop a 320-line F_6_-derived F_8_ recombinant inbred line (RIL) population, which was phenotyped in the field for IDC (Kocak, 2014). Thus, we plan to use this population to identify the factors controlling IDC tolerance and FluRE production.

## Author contributions

BW designed and conducted experiments, analyzed data, and wrote the manuscript. KA designed RNA-seq protocols and performed transcriptomic analysis. GG provided soybean germplasm and analyzed data. All authors edited and approved the final manuscript.

### Conflict of interest statement

The authors declare that the research was conducted in the absence of any commercial or financial relationships that could be construed as a potential conflict of interest.

## Supplementary material

Supplementary Figure 1 Diagram of phenylpropanoid synthesis pathway genes.

Supplementary Table 1 All genes that were differentially expressed by Fe or Bic treatments.

Supplementary Table 2 Selected differentially expressed genes.

Supplementary Table 3 Soybean homologs of Arabidopsis genes that are upregulated by microorganisms.

Supplementary Table 4 Thousand six hundred and twenty-six genes that were differentially expressed between genotypes and also were differentially expressed by Fe or Bic treatments.

Supplementary Table 5 Differentially expressed genes that correspond to previous QTL intervals.

## Abbreviations

IDC: iron deficiency chlorosis
FluRE: fluorescent root exudates
Bic: bicarbonate
FCR: ferric-chelate reductase.
