# Supplementary material for: Gene Expression Profiling of Iron Deficiency Chlorosis Sensitive and Tolerant Soybean Indicates Key Roles for Phenylpropanoids under Alkalinity Stress
Source: Front Plant Sci. 2018 Jan 19;9:10. doi: 10.3389/fpls.2018.00010 (PMC5780454; doi:10.3389/fpls.2018.00010)
Supplement: Supplementary Figure 1 — Diagram of phenylpropanoid synthesis pathway genes. [file Image1.PDF]

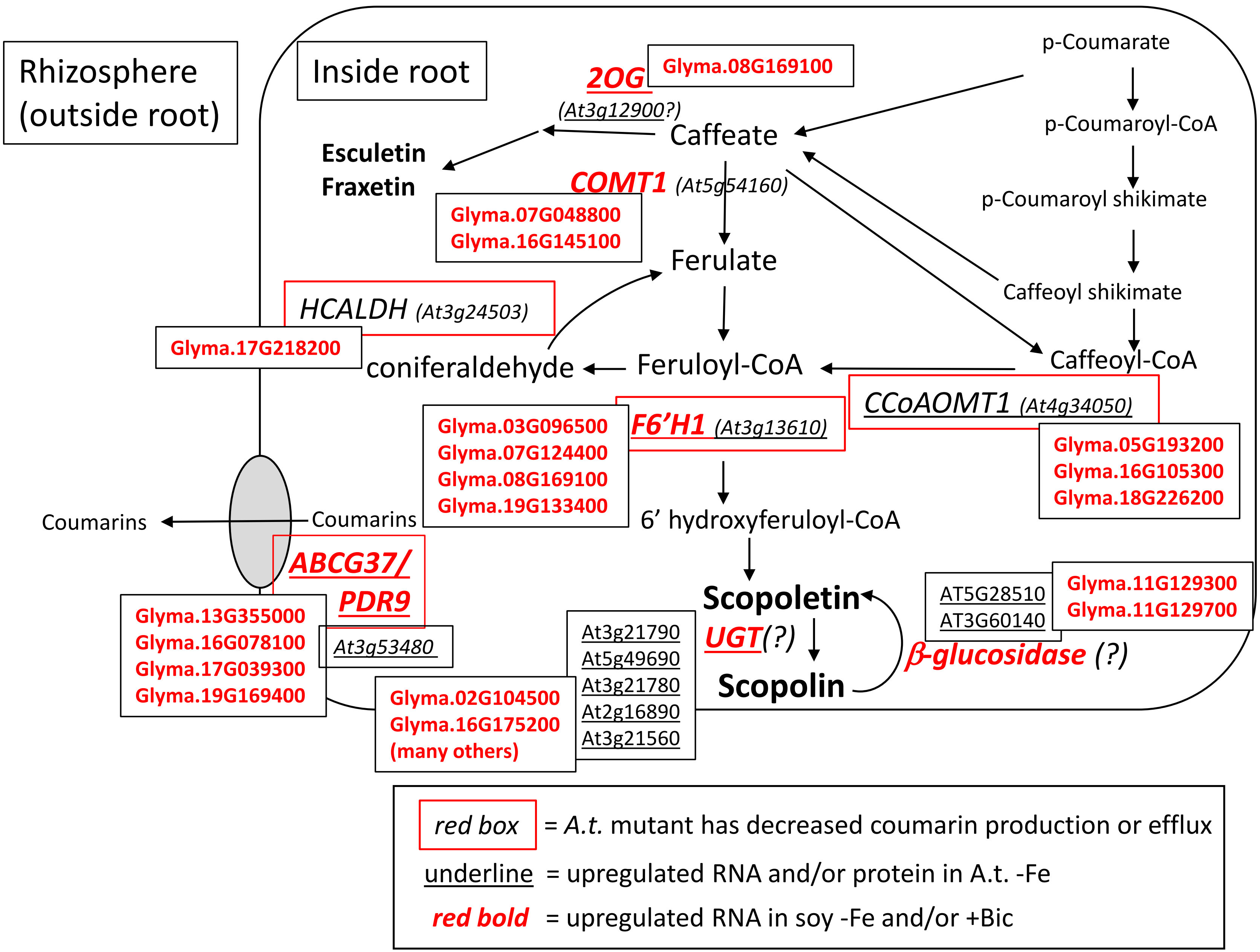
Rhizosphere (outside root)
Inside root
p-Coumarate
p-Coumaroyl-CoA
p-Coumaroyl shikimate
Caffeoyl shikimate
Caffeoyl-CoA
2OG
Glyma.08G169100
(At3g12900?)
Caffeate
Esculetin
Fraxetin
COMT1 (At5g54160)
Glyma.07G048800
Glyma.16G145100
Ferulate
HCALDH (At3g24503)
Glyma.17G218200
coniferaldehyde
Feruloyl-CoA
CCoAOMT1 (At4g34050)
Glyma.05G193200
Glyma.16G105300
Glyma.18G226200
F6'H1 (At3g13610)
Glyma.03G096500
Glyma.07G124400
Glyma.08G169100
Glyma.19G133400
6' hydroxyferuloyl-CoA
Coumarins
Coumarins
ABCG37/ PDR9
At3g53480
Glyma.13G355000
Glyma.16G078100
Glyma.17G039300
Glyma.19G169400
Scopoletin
UGT(?)
Scopolin
At3g21790
At5g49690
At3g21780
At2g16890
At3g21560
Glyma.02G104500
Glyma.16G175200
(many others)
AT5G28510
AT3G60140
Glyma.11G129300
Glyma.11G129700
β-glucosidase (?)
red box = A.t. mutant has decreased coumarin production or efflux
underline = upregulated RNA and/or protein in A.t. -Fe
red bold = upregulated RNA in soy -Fe and/or +Bic
